# Connecticut Valley and Massachusetts Dental Society

**Published:** 1888-11

**Authors:** Ira G. Baumgardner


### Reports of Society Meetings.

### THE CONNECTICUT VALLEY AND MASSACHUSETTS DENTAL SOCIETY.

### UNION MEETING, JULY, 1888.

REPORTED FOR THE "INDEPENDENT PRACTITIONER" BY IRA G. BAUMGARDNER, D. D. S.

[*Continued.*]

Discussion on Dr. Allan's paper on " The Germ Theory of Dental Caries, was opened by Dr. W. X. Sudduth, Philadelphia.

Owing to the lateness of the hour I shall make my remarks as brief as possible. Dr. Allan has presented a most excellent paper upon the subject and I have never seen the exhibit you have witnessed to-night excelled in this country. The photomicrographs are the finest it has ever been my pleasure to look at. Dr. Andrews has fairly outdone himself in their preparation. The value of such demonstrations as compared with drawings cannot be considered; you have seen thrown on the screen the shadows of the baccilli found in the dental tubuli. They are shadows of realities which if they did not exist could not have been photographed. The camera never invents or draws on its imagination. It tells the truth and deals only with facts. No chimera can find lodgment in the camera obscura. However strongly the connection of these micro-organisms with the etiology of decay may be denied, no man can go away from this hall to-night and say that their presence in the dentinal tubuli has not been clearly demonstrated. I cannot explain to myself how it is that the profession at large—medical and dental—has been so averse to accepting the " germ theory." It is true that its advocates promulgated theories that were radically opposed to those which have held sway for many years. The opposition may, perhaps, have been due to this fact. It has been steadily gaining ground, however, until to-day it is firmly rooted in all our literature, medical and dental. The criticisms hurled against it have only tended to establish it all the more firmly. One significant fact might be mentioned in this connection, and that is that all the young men who have come into the profession within the last decade have accepted it with but very few exceptions. Those who hold out the most strenuously against it are the older members of the profession who were educated under the old regime and who have found it difficult to shade off the shackles of prejudice, and accept the only theory that has been advanced to explain the heretofore unexplainable in medicine. The "Germ Theory of disease" has done more to remove the "Ifs" from out our pathway, and make an exact science out of medicine than all the other work that has been done put together. Dr. Miller's theory of the formation of cavities by the action of a digestive ferment upon the basis substance of dentine has been the only theory ever advanced that explains the formation of cavities. At one blow he swept away all the speculations of past years regarding the etiology of decay. There is a vast difference between speculation and theory. A theory must have some basis in fact while speculation may soar aloft untrammelled by sordid reality.

I would say more, were it not for the lateness of the hour. I hope, however, that the subject will be called up in the morning.

MORNING SESSION, JULY 12, 1888.

Discussion Continued.

*Dr. W. H. Atkinson*, New York City—I have nothing but commendation to offer for Dr. Allen's paper. It was clean-cut and straightforward, but he said it was a collection of what he had read on the subject more than what he had done. Other than the making of specimens and his interpretations, the paper is exactly a reversed truth. He said that the secondary dentine that so uniformly follows the insertion of fillings in decayed cavities in the teeth settled the question as to the non-inflammatory process of the reparative activity that forms secondary dentine. Had he known the influences operating to produce it he would have seen it was the exact initiative of the process, which, when arrested just before it becomes pronounced inflammation, becomes "reparative" inflammation. The difference is that it has not arrived at the true inflammation.

He also said he had the greatest pleasure in endorsing Dr. W. B. Miller's statement regarding the identity of artificial decay to that produced in the mouth according to the natural process. There is not one of those cases that cannot be discovered in an instant as to which was natural and which artificial. In every one that was artificial the micro-organisms followed the line of the tubules without striking into the consolidated inter-tubular substance that we call the basis substance of the dentine.

*Dr. M. L. Rhein*, New York City—I would like to ask your interpretation of the pipe-stem series.

*Dr. Atkinson*—It strikes me that we do not know the cause of their formation. It has not been proven that microbes get into the pipe-stems. What is it that acts against the lime salts? Let him that says it is an acid prove it. You saw that beautiful specimen of a lateral incisor, cut thirty years ago by Dr. G. S. Allen, in my laboratory in Cleveland, O. Have we got ahead of the instruction that is in that slide yet? Not by a good deal, and we use it to-day to show that what has been said within the last ten years is not so new as it would appear

I have shown you clinically in years past cases of alveolar abscesses that were pumped full with wood creosote, so that the fistulous opening was whitened, and the roots and crowns filled, and they remain sound until to-day. One case in particular I remember of a man affected twenty-four years with a discharging fistula—left central incisor. In four weeks, with two or three applications, it was cured. Even if we find difficulty in knowing what these microbes are, we do know how to squelch them.

Prof. Mayr's paper was almost perfect. It did not have a single ambiguity, and was so luminous that all we have to do is to take our little torches and light them at the ignition of his statements to make us see what there is in starry radiance in atoms. The cadaveric poison in ptomaines, he said, must have the light of ammonia. If we were to comprehend the chemistry of it, it would so occupy us that we would not do anything but study chemistry all the remainder of our lives.

*Dr. C. N. Pierce*, Philadelphia—Let me thank you, Mr. Chairman, for the opportunity of expressing my gratification in listening to the two papers read, by Prof. Mayr and Dr. Allen. I came to Boston almost exclusively to hear them.

Dr. Sudduth, in his remarks, said that he could not see why people were so slow in accepting the "germ theory of disease." I want to say just one word, and speak of an instance or two among many that I have recognized as reasons for the slowness with which the theory has been accepted. In a scientific body in Philadelphia, a gentleman came before the association with a paper on a poisonous substance supposed to be secreted by the kidneys. The paper was well illustrated; but upon investigation, it was found that the article that had been examined microscopically was not what it was thought to be at all. The servant had thrown out the original urine, and to cover up his carelessness had substituted something else. In another instance an elaborate paper was written on the subject of a new vomit—low forms of life. A subsequent examination which was directed showed that the patient's bed was close to a window near which grew an ivy vine, and what the physician had found in the vomit in large quantities was the stellate particles of the ivy leaf. The patient had chewed these, and they made him sick. This liability to error which is known to exist is one of the reasons why people have been slow in accepting the germ theory of disease.

I have one criticism to offer on the delightful paper and exhibition made by Dr. Allen last evening. In his desire to impress upon the audience the influence of microbes or low forms of life in the production of dental caries, he was disposed to ignore what we term predisposing causes, or an influence—constitutional or local—that may prevent or modify the progress of the disease. I do not think it was intentional; but it might appear so. He showed us a beautiful specimen of a lateral incisor, upon the palatine or lingual surface of which was a deep fissure, running down from the basilar ridge. Such a fissure in the mouth of a young miss about to graduate from school, striving for prizes, etc., with a stimulated nervous system, would go to the pulp in less than three months. Suppose that fissure now in a tooth in the mouth of a mature individual, with good nutrition and functions, in which case it might remain there without material change for three years, and not endanger the pulp chamber; so that nutrition and function have modifying influences, and must always be taken into account in the progress of dental caries.

Again, suppose it in the mouth of a mother or a person about to become a mother, where every energy is taken in building up the child—we have the same result: decay extending into the pulp in three months; where, in the normal condition, when all the energies are properly protected and the system is receiving sufficient nutrition, it may remain there for years without further progress.

The beautiful illustration of the transverse sections of the tubuli showed that in certain tubuli we had masses of a low forms of life making great destruction; while in others none were present. They do not attack so much the more indestructible, but seek the weakest portions; and that is why they enter the tubuli. They go there because there is organic matter that offers them a pabulum. Where we have no pabu.um, we have no life. Life is always present where there is anything to sustain it.

Another word regarding Dr. Atkinson's remarks. I value him as a teacher, and know him to be possessed of many commendable attainments; but he made a remark this morning that is a little unjust. He referred to the illustration shown last night, where the bacilli were shown in the tubules, and asked, How do we know they are low forms of life? We hardly think Drs. Allen, Sudduth and Andrews would come before this body or any other and state that in those tubuli we had low organisms, unless they were fully convinced of their identity.

When I came to the city on Tuesday morning, almost the first gentlemen I met said, "Well, Pierce, have you been converted yet?" alluding to the inference that I was not a believer in the germ theory of dental caries. I am a firm believer in the presence of low forms of life, whereby dental caries exists; and I know, from the statements of Dr. Allen, Miller and others, that artificial caries has been induced by the presence of these low forms of life. I am a firm believer in the fact that dental caries cannot progress without these low forms of life; but what I desire to say is this: I want it recognized that there is a power that may anticipate or even overcome them; and that where you have an influence brought to bear, either through systemic or local conditions, functional or otherwise, they may be overcome; may have their action prevented, and that their influence is greater where there is a deficiency in recuperative force. Then it is that these organisms have the power to do the greatest mischief.

*Dr. M. L. Rhein*—I was very much interested in the admirable illustrations which we all witnessed last night, and I find myself very much in the position of Dr. Pierce. I believe that the germs play an important part in the etiology of dental caries, but not to the extent that the author of last night's paper wants us to believe. As I understood him, he places the entire cause of caries upon micro-organisms, and allows nothing else to enter into the consideration. This is the antagonizing point that Dr. Pierce has taken up, and it is that which met me at every stage of the illustration last night. All of the illustrations tended to convince me that there were other points entering into the cause of the caries.

I want to protest against the point laid down by Dr. Allen that we know the cause of dental caries, and that the "Germ Theory" explains everything. There is to my mind a great deal more to be explained than what was explained last evening.

There was one more point in the paper that I oppose, and that is the statement that the enamel is non-vascular. The illustrations presented last evening distinctly showed that vascular fibrils penetrate the enamel. We know that the enamel bears the same relation to the dentine, in one sense, as the epithelial layer of skin does to the true derm. That there is a constant waste and repair in the epithelial layer of the skin is not questioned, and yet we find no evident traces of vascularity; but that does not prove that directly underneath there is not vascular tissue. In some teeth I have seen under the microscope very great vascularity under the enamel.

*Dr. W. X. Sudduth*—If any one has a right to speak authoritatively in regard to this matter, it is surely those who have worked experimentally on the subject, who have actually cultivated and studied these germs, and who for years have given it their attention. There are several men in the profession who have done this. They desire to go hand in hand with men of clinical experience. Whenever I find that my scientific work does not agree with the daily experience of those who work at the chair, then I immediately doubt my own conclusions and not those of the clinician. If we cannot practically demonstrate our theories to you, and harmonize them with your experience, then the probabilities are that we are at fault.

There is no other way that a cavity can be made but by micro-organisms. The action of acids will not produce cavities. It is only by the action of a digestive ferment that they are formed. Every tooth contains basis substance, and it is not only by the dissolution of the lime salts ; but by the dissolving out of the basis substance that cavities can be formed. Those who oppose the germ theory have never given any plausible opinion as to how cavities are formed.

In regard to the question of natural and artificial caries, artificial caries is identical in its histological appearance with natural caries and no one can tell the difference.

*Dr. Atkinson*—I can tell.

*Dr. Sudduth*—I will take a number of slides of each, and guarantee that Dr. Atkinson or no one else can tell the difference.

The micro-organisms follow in the line of the tubules, and go from one to the other through the lateral branches as was so beautifully shown last evening. Dr. Atkinson criticised what I said regarding their physical appearance. He misapprehended me. I said that we did not have to depend entirely upon the microscope. We can, in many instances, differentiate the several varieties by their physical appearance in the test tube. It is not absolutely necessary to use high powers. Different varieties have essentially different physical characteristics, such as variations in color, as was demonstrated last evening. Others differ in the rapidity with which they liquefy gelatine; still others in the form of the growths, or the shape of the colonies on plate cultures. To understand these different expressions and to a full understanding of the subject it is essential that one have more or less practical experience in the laboratory. One conspicuous fact is brought out in all these discussions and that is that the opponents of the "Germ Theory" use the terms, "I think," "I believe," etc., showing their lack of positive knowledge on the subject, while those of us who have worked on the subject have at least a basis of fact to go upon. It is an eminently practical subject and one capable of demontration, and we gave you a demonstration of the presence of the germs in the dental tubuli last evening.

*Dr. Werner*, Boston—I desire to call the attention of the speaker to the resistive power in the tooth while it is alive.

*Dr. Sudduth*—In my opinion the only resistence offered by the tooth is expressed in the pulp chamber in the development of secondary dentine. The tooth, except for this, is a perfectly passive agent. I have given this subject more attention than any other phase of caries. It has been held that a zone of resistance is thrown out by the tooth in advance of the forming cavity. No experiments have ever been made that substantiated the statement; it therefore remains the merest speculation. On the other hand the experiments of Dr. Miller show that there is probably no increase in the amount of lime salts found in the, so called, zone of resistence. The chances for error are so great that I very much doubt if the question will ever be satisfactorily settled by chemical analysis. I think the best answer to the question is found in the fact that under similar circumstances, in the same mouth, that teeth with devitalized pulps do not decay any more rapidly than do teeth in which the pulps are alive. Some may dispute this latter statement, but I think that the further fact that there is a growing sentiment in the profession that pulps are not as valuable as they were once considered, bears me out in the statement. This, in my opinion, is a sure indication that clinical experience finds little or no difference as regards the liability of pulpless teeth to decay over those containing live pulps.

EVENING SESSION. THURSDAY, JULY 12, 1888.

Close of discussion on Dr. Allen's paper.

*Dr. Geo. S. Allen*, New York City—If you remember I said that Dr. Miller had found, as have others, bacteria in the dentinal tubules of carious teeth. I told you that he had obtained a pure culture of these bacteria, and had continued them for successive generations; that into this pure culture he had placed pieces of some healthy teeth, adding to it a fermentable mixture, also sterilized; and that artificial caries had been produced simulating absolutely in all physical characteristics that of natural caries. In the artificial caries the bacteria from the original pure culture were found in the dentinal tubules. What could be more conclusive than that?

Dr. Sudduth also called your attention to the fact that Dr. Miller had gone one step further than most workers in mycology. He has not only produced artificial caries by the action of bacteria; but has found out what the "ptomaine" of the carious fungus is, namely, lactic acid. In the demonstration I gave you on the screen of artificial and natural caries, I am positive that no microscopist could tell them apart. It is simply impossible. The assertion made by Dr. Atkinson to the effect that from simply looking at these views he could tell the difference is something that passes my understanding. I think it is time that in our dental meetings—when a scientific question comes up, that it should be discussed in the spirit in which it is presented—not simply by making dogmatic assertions. This subject is now so firmly fixed in the minds of all thinking men that it is not likely to be offset by such a line of argument.

*Dr. R. R. Andrews*, Cambridge, Mass—Dr. Allen spoke of a case where pieces of dentine were subjected to the action of the fluid. Will he kindly tell us more about the experiment?

*Dr. Allen*—The experiment Dr. Andrews speaks of was this: Into a tube containing a pure culture of the bacteria of dental caries pieces of sound healthy teeth which had been previously sterilized were placed. The mixture used was a beef extract, to which 2 per cent. of cane sugar had been added, so as to make it a fermentable mixture. In this tube containing the sterilized beef extract and sugar, and the sterilized teeth, with the pure cultures of bacteria, these experiments were made. The tubes were sealed up with a cotton plug, sterilized, and the whole placed in a thermostat which consists of an oven in which the temperature can be evenly maintained for any length of time. At the end of a week the pieces of sound healthy teeth placed in this mixture were so fully decalcified that they could be bent readily, showing that a large proportion of the lime salts had been extracted. There is nothing that will take lime salts out of tooth substance but an acid. It is absurd to talk of breaking up the lime salts by any other way than by the action of some acid. At the end of two weeks the pieces of teeth commenced to show signs of disintegration, and in the course of two or three months the whole tooth had disappeared. These experiments show that there were two agents, at least, namely, an acid which had dissolved out the lime salts, and a peptonizing ferment, which, when the lime was dissolved, had broken down the basis substance of the tooth-animal matter, which is composed of proteids—calcoglobulin. This experiment Dr. Miller repeated frequently, and there is no question about its value.

*Dr. Pierce*—The doctor states that the solution used had been previously sterilized, was the object of sterilization, intended to keep out all other organisms except the one desired.

*Dr. Allen*—That is correct.

*Dr. Pierce*—If I understand you, the decomposition of the lime salts was through an acid product of the germs placed in the liquid.

*Dr. Allen*—Yes, the question that Dr. Andrews asked led to the statement of the fact that there was, in addition to the acid present, a peptonizing ferment that carried on the work that the acid left unfinished.

In conversation with one or two dentists this afternoon, and also in listening to my friend Dr. Pierce this morning, the idea came to me that I might say a word in regard to the indirect causes of decay, which was alluded to by Dr. Pierce and Dr. Rhein of New York city. Dr. Miller has investigated fully all these indirect causes of decay, so called, such as fissures, crowded arches, etc., and imperfectly developed teeth. Dr. Miller draws a line of distinction, however, between cause and conditions favorable to decay. The latter are not causes of decay. Dr. Miller appreciates all these points fully and places stress upon the one fact that the active cause of the decay is bacteria, and by the action of these alone he has produced artificial caries showing all the similar characteristics of decay occuring in the mouth.

Subject passed.

Discussion on paper read by Dr. Cheney on " The Treatment of Pulpless Teeth," published in October number.

*C. N. Pierce*, Philadelphia—This paper was of especial interest, conservative, and markedly correct in the course of treatment pursued. The methods of filling pulp cavities, however, adopted by different members of the profession, varies in different localities, and it is possible that the various forms of treatment may be equally satisfactory.

There is no doubt that the pulp chamber, once thoroughly cleaned, and made entirely free from the debris resulting from dead pulp or food, and well-filled should, in a majority of cases, remain entirely healthy—healthy as regards any subsequent disturbance that will injure the pericementum. We have, however, a great many patients that are predisposed to irritability of tissue, and the fact that we have disturbed the normal circulation of the tooth, by diverting the flow of blood from the pulp to the pericementum, produces a pericementitis on very slight occasion. The pericementum thus often becomes the seat of irritation, and very often of inflammation. Because a tooth becomes affected with pericementitis after the pulp has been destroyed, and the root filled, is no evidence that it has not been done in a proper manner, or as perfectly as possible. We may find in one patient a condition that would produce perfectly healthy results, while in another patient, owing to abnormal conditions, bad results may unfortunately occur simply through the different constitutional tendencies of the patient, so that it is not safe to condemn a method of practice, or a condition that exists because the patient is suffering from pericementitis.

In my practice in filling pulp chambers, whether pulps devitalized by myself or pulps dead for some time, I prefer to use oxide of zinc mixed with chloride of zinc in preference to gutta-percha, and I like it because of its thoroughly antiseptic property. I fill all the roots with what might be termed "oxychloride of zinc," mixing it in the form of a thin paste. Gutta-percha is not a therapeutic material; it is a mechanical agent. In many cases we want more than the filling of the root—we went an agent for filling that has antiseptic properties, and those we get in the chloride of zinc; and I get better results with it than with gutta-percha.

When I recall the years of experience of many in practice, and see the large number of teeth that are saved—saved for years without the pulps—I think it a pretty good record for pulp devitalization, and while I feel that the tooth is better with a live pulp, if it is possible to save it—when the pulp is exposed, and beyond the hope of salvation from local or constitutional conditions, I consider it is good practice to devitalize it. The results of such practice encourage us in so doing.

The question of filling or treating teeth is one that interests every dentist, and one that comes home to every practitioner, and we ought not to be at a loss for any amount of expression on this subject; for every one has cases to treat, and each one's experience is worth something.

*Dr. C. T. Stockwell*, Springfield, Mass.—I would like to ask Dr. Pierce if the chloride of zinc has anything more than a momentary antiseptic quality, if after it is used it does not become inert?

*Dr. Pierce*—When I place oxychloride in the root I place it in very thin, and do so by wrapping my instrument with some very short fibres of cotton, and pass it in, using a pressure sufficient to cause the fluid to thoroughly saturate the walls of the pulp chamber. By so doing, if any pulp tissue has been left in the canal, it becomes saturated with zinc, and is protected from decomposition.

I sometimes find that cavities which have been filled with gold or gutta-percha, some months afterward become offensive, because gutta-percha in the pulp chamber has to a certain extent become disintegrated; whereas with chloride of zinc we have no disintegration.

Subject passed.

Discussion on Dr. Fillebrown's paper, read by Dr. Thomas Fillebrown, of Portland, Me., on "The Relations of the Teeth and Palate to Vocalism."

*Dr. C. N. Pierce*, Philadelphia—To criticize the paper is beyond my capacity. I can only say that its conception, its thoroughness and the manner of presenting it have been almost perfect. I would simply say that, in regard to articulation, I should like to have time to call the attention of the audience to the importance of the teeth in certain sounds; because it is a matter of great importance to us regarding the replacement or retention of those organs. Many of us, oftentimes, greatly impair the speech of our patients by the awkwardness or imperfectness with which we insert dentures; oftentimes by our means of correcting certain defects and leaving spaces between the teeth, patients complain that they are biting their lips or cheek, when it is simply their effort, with the lips or cheeks, to supply the office of the lost tooth in order to make the cavity essential to articulation. I have given more attention to this feature of the question.

*Dr. Charles C. Barker*, Meriden, Conn.—I would like to ask Dr. Fillebrown the function of the uvula. That falls under the head of oral tissues. It touches a point I am very much interested in. I would like to ask him the function of the uvula in the production of tone.

*Dr. Thomas Fillebrown*—My researches have not gone far enough for me to know what the office of the uvula is; persons *seem* to speak just as well after it is gone as before. I think that it has its place. I am a firm believer that the Creator never made anything which was not needed. The office of the spleen is not well understood; yet it is an essential part of the body. So with regard to the uvula.

*Dr. Ottolingui*—What part do the tonsils play?

*Dr. Fillebrown*—If they are too large they impede the voice. They then form a mechanical obstruction.

While I recommend the removal of a portion of enlarged tonsil; yet I would not remove any considerable portion. After the operation absorption will soon take care of the rest. In regard to the obturator, the action of the muscles would draw down in front of the hinged end, and will throw it back. The obturator should be long enough to come down to the bottom of the uvula, so that when it springs back it will strike against the basilar process. I reached that conclusion only a short time ago. I made two obturators for a patient, and she had trouble with them. I studied the matter, and finally made a third one, and made it spring up; and it seemed that her basilar process was lower than common. I made one for her so that when it sprang back it would hit the basilar process, and she has improved wonderfully.

I had a case of a young man studying for the priesthood. Although very bright, his deformity shows the incongruity of his selection of a profession. He has a cleft clear up to his hard palate, and could not talk well enough to be heard. I have helped him out wonderfully. I made him an obturator, and he has labored hard in the practice of vocalism; and where he formerly had a strident voice, it is now considerably softened, and he can speak the mutes without difficulty, and gets his vowels in good shape.

*Dr. H. A. Baker*, Boston—This is a subject I am somewhat interested in. I do not want to criticize Dr. Fillebrown's paper, except in one statement, and that is in speaking about the nose—"he talks through his nose." Now all of you can try this. Close your nose and you can give all the alphabet but m and n. He is perfectly correct in regard to articulation and resonance. They are two different things. A person with cleft palate can articulate as well as any one; but resonance is so indistinct that he can hardly be heard.

This calls to mind a case I had a number of years ago—a case where a polypus had been taken out of the nose, and there was an immense cavity. I put in an obturator, and the patient could articulate as well as any one, but the resonance was very bad. As many of you know I have had experience in some one hundred cases covering a period of nearly twenty years. He speaks about the "ball obturator." I think one of my first experiments was an obturator similar to what he has shown on the board, almost like it, with the exception that it had a piece of flat rubber stitched on to articulate with the pharyngeal wall; but I have had better success with others. I think I have something like ten cases in which I do not think a person in the audience could detect any deficiency in the articulation.

