# A Correction

**Published:** 1889-01

**Authors:** 


### A CORRECTION.

Dr. M. L. Rhine desires us to say that in reporting his remarks on Dr. Allan's paper at the Boston meeting, we make him say "vascular" fibrils when "living" fibrils was intended. The error occurs on page 584 of the November number in the sentence, "The illustrations presented last evening distinctly showed that 'vascular' fibrils penetrated the enamel."

